# Improving lung cancer survival; time to move on

**DOI:** 10.1186/1471-2466-12-77

**Published:** 2012-12-13

**Authors:** Marlies E Heuvers, Joost P Hegmans, Bruno H Stricker, Joachim G Aerts

**Affiliations:** 1 Department of Respiratory Diseases and Tuberculosis, Erasmus Medical University Center, Rotterdam, The Netherlands; 2 Department of Epidemiology, Erasmus Medical University Center, Rotterdam, The Netherlands; 3 Department of Internal Medicine, Erasmus Medical University Center, Rotterdam, The Netherlands; 4 Department of Medical Informatics, Erasmus Medical University Center, Rotterdam, The Netherlands; 5 Department of Respiratory Diseases and Tuberculosis, Amphia Hospital, Breda, The Netherlands

**Keywords:** Lung cancer, Survival, Lung cancer screening, Immunotherapy

## Abstract

**Background:**

During the past decades, numerous efforts have been made to decrease the death rate among lung cancer patients. Nonetheless, the improvement in long-term survival has been limited and lung cancer is still a devastating disease.

**Discussion:**

With this article we would like to point out that survival of lung cancer could be strongly improved by controlling two pivotal prognostic factors: stage and treatment. This is corresponding with recent reports that show a decrease in lung cancer mortality by screening programs. In addition, modulation of the patient’s immune system by immunotherapy either as monotherapy or combined with conventional cancer treatments offers the prospect of tailoring treatments much more precisely and has also been shown to lead to a better response to treatment and overall survival of non-small cell lung cancer patients.

**Summary:**

Since only small improvements in survival can be expected in advanced disease with the use of conventional therapies, more research should be focused on lung cancer screening programs and patient tailored immunotherapy with or without conventional therapies. If these approaches are clinically combined in a standard multidisciplinary policy we might be able to advance the survival of patients with lung cancer.

## Background

Lung cancer is the leading cause of cancer-related death worldwide. Approximately 85% of all cases of lung cancer are non–small cell lung cancer (NSCLC). The 5-year survival of this aggressive disease is only 16% [1]. One of the reasons for this extremely poor survival is that most lung cancer cases are diagnosed at an advanced stage due to the relative lack of clinical symptoms during early stages. Metastatic NSCLC is currently an incurable disease for which standard chemotherapy provides only minor improvement in overall survival. In addition, less than 30% of patients with advanced-stage NSCLC have a response to platinum-based chemotherapy, the most commonly used first line treatment at this stage of the disease [2].

During the last decades, advances in diagnostic and therapeutic approaches of this devastating disease have been made, however, long-term survival rates have hardly changed in the past 50 years [3]. Therefore, new approaches are required.

## Discussion

Survival of lung cancer could be strongly improved by controlling two pivotal prognostic factors: stage and treatment. Early stages of lung cancer have a better prognosis; thus early diagnosis of lung cancer by screening programs is one way that leads to a reduction in lung cancer mortality. However, given the high chance of tumor recurrence, even alleged early stage NSCLC patients with adequate surgical resection can have undetectable metastases at diagnosis [4,5]. It is known that adjuvant chemotherapy can reduce these metastases; nevertheless, in 24% of the patients metastasis occurs after adjuvant chemotherapy [4]. Therefore, besides lung cancer screening programs, an additional approach next to the conventional therapy must be developed to tackle lung cancer. In recent years it has been established that the immune system plays an important role in carcinogenesis and makes an essential contribution to the anti-tumor effects of traditional therapies. Modulation of the patient’s immune system by immunotherapy either as monotherapy or combined with conventional cancer treatments offers the prospect of tailoring treatments much more precisely and could lead to a better response to treatment and overall survival of NSCLC patients.

Taken together, when early diagnosis by screening programs and patient-tailored immunotherapy are combined in a standard multidisciplinary policy for NSCLC treatment, we might be able to advance the survival of patients with early stage lung cancer. We will discuss both topics and their role in improving lung cancer survival below.

### Lung cancer screening

Multiple randomized trials have investigated the effectiveness of lung cancer screening and it is shown that lung cancer can be identified at an early stage with detection rates varying between 40-66% [6,7]. The survival rates of lung cancer patients diagnosed in screening programs are very high; 5- and even 10-year survival rates close to 90% can be achieved [8,9]. The largest lung cancer screening trial [10] recently showed that screening of high risk persons is very effective in reducing the mortality from lung cancer. Persons with more than 30 pack-years (PY) and aged between 55 and 74 years at time of randomization were included in this study. They found a relative mortality reduction of 20% when this high-risk group is screened with a low-dose computer tomography (CT) scan compared to chest radiography [10]. However, this is probably an underestimate, as the mortality reduction was measured at the time of closure of the trial. The introduction of low-dose multi-detector CT has led to important advantages, such as advanced scan speed, better spatial resolution, and the capacity to reconstruct multiple series from a single data acquisition. Before public policy recommendations are crafted, there are major concerns in lung cancer screening such as the effects of false positive findings, lead-time bias, the impact of overdiagnosis, and the generalizability of the results [11].

Another important aspect that should be considered in generalizing the results of screening studies are the therapeutic options for patients with a positive screening, as lung cancer treatment is an important prognostic factor. In developed countries, lung cancer patients are treated with surgery, chemotherapy, and radiotherapy. In recent years, peri-operative mortality has decreased by the introduction of video assisted thoracoscopy (VATS) and better peri-operative management [12]. Early stage patients who are not eligible for surgery are frequently treated with radiotherapy with curative intent. Novel radiotherapy techniques, such as stereotactic ablative radiotherapy, show local control rates of 90% or more for stage I NSCLC [13]. Adjuvant chemotherapeutic regimens have been shown to increase survival especially in resected patients with stage II and IIIA disease [14]. These regimens are expensive and therefore the results of the published screening trials can only be applied to the selected group of individuals in countries with well-developed health care systems with a quality comparable to the US.

### Adjuvant immunotherapy

Treatment of lung cancer is currently based on the patient’s clinical signs and symptoms, tumor stage and subtype, medical history, and data from imaging and laboratory evaluation. Until now, most cancer research is focused on therapies based on tumor characteristics to improve the prognosis of NSCLC, as cancer has long been considered as a cell-autonomous genetic disease. However, the sobering outcome of current NSCLC therapy has shifted the attention to combining adjuvant treatment approaches.

Recent experimental findings and clinical observations have led to cancer-related immune inflammation being acknowledged as a new hallmark of cancer [15-17]. Evidence that the immune system of the host can influence cancer incidence, cancer growth, response to therapy, and the prognosis of the disease, is growing [18]. Therefore it was thought that conventional therapy combined with immunotherapy based on a pretreatment profile of the immune system of the host could be a valuable tool to increase the survival of early stage NSCLC [19].

Cancer immunotherapy attempts to activate the host’s immune system to recognize and destroy the residual lung cancer cells that conventional therapy misses. Immunotherapy can be divided into two main types: passive and active immunotherapy [20,21]. The most common form of passive immunotherapy is monoclonal antibody therapy [20]. It makes use of antibodies that have been produced in vitro and can bind to specific cell surface proteins that can influence tumor growth [22]. However, there will only be a response of the immune system during the time the antibody is present in the body. Ipilimumab (anti-CTLA-4), bevacizumab (anti-VEGF), and anti programmed death (anti-PD-1) or anti-PD ligand 1 (Anti-PD-L1) are examples of passive immunotherapy that could be useful in NSCLC [23-26].

Ipilimumab blocks the negative cytotoxic T-lymphocyte antigen (CTLA)-4 that enhances T-cell responses to tumor cells, leading to effective immune responses. For NSCLC, ipilimumab is now in phase II development [24], but it is already approved by the US Food and Drug Administration (FDA) for the treatment of unresectable or metastatic melanoma [24,27,28]. Studies show that the two- and three-year survival rates in ipilimumab-containing treatment arms in metastatic melanoma patients are almost twice as high as in the non-ipilimumab-containing treatment arm.

Bevacizumab is an antibody that neutralizes the vascular endothelial growth factor (VEGF) ligand. As a result, it will inhibit angiogenesis [29]. Moreover, research has shown that adding bevacizumab to chemotherapy is associated with afferent vascular dilatation and efferent vascular constriction of tumor vessels that may help concentrate chemotherapy at the tumor site. Bevacizumab combined with taxane-platinum chemotherapy is the first approved antiangiogenic agent for cancer therapy that showed increase of progression-free survival and overall survival in first-line treatment of stage IV NSCLC [29,30]. Recently, data have been published on the immunogenic effect of VEGF. VEGF seems to be involved in a number of mechanisms negatively influencing the immune system; it makes dendritic cells more tolerogenic, and induces myeloid derived suppressor cells. Adding bevacizumab prevents immunotolerance and could thereby contribute to a better survival of lung cancer [31,32].

Two other recently described antibodies that could play important roles in passive immunotherapy are anti-PD-1 and anti-PD-L1 [25,26]. PD-1 is a co-inhibitory receptor on activated T-cells that plays an important role in immunosuppression. PD-L1, the ligand of PD-1, is expressed on cancer cells and is involved in negative regulation of immune responses, as they increase apoptosis of T-cells and inhibit CD4 and CD8 T-cell activation [25,26]. Inhibition of the interaction between PD-1 and PD-L1 can improve T-cell responses and mediate antitumor activity. Recent studies show that in NSCLC the objective response rates to anti-PD-1 and anti-PD-L1 are 18% and 10% respectively [25,26]. Blockage of both receptors induced durable tumor regression and prolonged stabilization of the disease. These findings confirm that the pathway between PD1 and PD-L1 could play an important role in therapeutic intervention and that it causes an increase in survival of lung cancer patients.

Active immunotherapy tries to persuade and boost immune effector cells *in vivo* against tumor cells through the administration of immune mediators capable of activating the humoral (antibodies) and cellular (T cells) immune system [33]. Therefore the duration of this broad response persists for a long time, because of the immunologic memory and it is less prone to antigen mutational responses [33]. Currently, multiple trials are investigating the effectiveness of different lung cancer vaccines [33-36]. In 2001, one of the first synthetic lung cancer vaccines showed that 16 out of 65 patients had an immune response after vaccination, and the median survival time was more than doubled (30.6 months, instead of 13.3 months in controls) [37]. After that, other tumor-antigens vaccines, such as Wilms tumor antigen-1 and IDM-2101 were tested and showed immunological responses and prolonged survival in patients with lung cancer [21,36]. Next to synthetic vaccines there are trials that test dendritic cell (DC) vaccines [34,38,39]. In DC vaccines, tumor associated antigens are used to load immature autologous DCs. These DCs are injected into patients to stimulate antigen-specific immune responses in lung cancer patients. Different studies have shown biological activity of DC vaccines and phase I and II trials report that a group of lung cancer patients had therapeutic benefit [34,39,40]. Nevertheless, until now, reports about clinical applicability are anecdotal.

Other examples of active immunotherapy in lung cancer are natural killer (NK) cell transfer and adoptive T cell transfer [41,42].

As described above, recent literature provides evidence for many potentially useful immunotherapy combinations. However, these therapies show drastic antitumor responses in only small subsets of patients. Currently, there is lack of predictive biomarkers to rationally choose combinations of immunotherapy for individual patients that benefit from these therapies. Therefore, it is necessary to further elucidate the mechanisms that are responsible for clinical benefit in small groups of patients and identify relevant pre-treatment biomarkers that distinguish responders from non-responders. This patient-tailored treatment approach is able to redress the balance towards efficacious antitumor responses that can improve the overall survival for more patients.

Taken together, passive and active immunotherapy might have an important adjuvant role in early stage NSCLC by consolidating responses to conventional therapy and thereby leading to increased lung cancer survival rates. However, further research in this field is warranted to improve these therapies and to define subsets of responders.

## Summary

During the past decades, numerous efforts have been made to decrease the death rate among lung cancer patients. Nonetheless, the improvement in long-term survival has been limited and lung cancer is still a devastating disease [3].

Since only small improvements in survival can be expected in advanced disease with the use of conventional therapies, more research should be focused on early stage lung cancer. Combining lung cancer screening programs and patient tailored immunotherapy with or without conventional therapies should be further explored. If these approaches are clinically combined in a standard multidisciplinary policy we might be able to advance the survival of patients with lung cancer.

## Abbreviations

NSCLC: Non–small cell lung cancer; PY: Pack-years; CT: Computer tomography; VATS: Video assisted thoracoscopy; FDA: US food and drug administration; VEGF: Vascular endothelial growth factor; DC: Dendritic cell; NK cell: Natural killer cell.

## Competing interests

The authors declare that they have no competing interests.

## Authors' contributions

All authors were major contributors in writing the manuscript. All authors read and approved the final manuscript.

## Pre-publication history

The pre-publication history for this paper can be accessed here:

http://www.biomedcentral.com/1471-2466/12/77/prepub
